# Technical review by the Brazilian Society of Surgical Oncology and the Brazilian Society of Head and Neck Surgery on hierarchical coding in thyroid surgery

**DOI:** 10.1590/0100-6991e-20250024-en

**Published:** 2025-12-23

**Authors:** CARLOS EDUARDO SANTA RITTA BARREIRA, FERNANDO LUIZ DIAS, TERENCE PIRES DE FARIAS, LUIZ PAULO KOWALSKI, IZABELLA COSTA SANTOS, JOSÉ GUILHERME VARTANIAN, ANDRÉ FERRARI BELTRÃO, ULLYANOV BEZERRA TOSCANO DE MENDONÇA, BRUNO SIMAAN FRANÇA, CARLOS CHONE, ANTÔNIO VITOR MARTINS PRIANTE, GUILHERME DE SOUZA SILVA, PETERSON FASOLO BILHAR, PAOLA ANDREA GALBIATTI PEDRUZZI, RAFAEL DE CICCO, STENIO ROBERTO SANTOS, DORIVAL DE CARLUCCI, ALINE DE OLIVEIRA RIBEIRO VIANA, CHRISTIANA MARIA RIBEIRO SALLES VANNI, MARINA AZZI QUINTANILHA, ADDILIS FONTE, RICARDO MAI ROCHA, ANDRÉ POVOA MIRANDA, MARCELO CAVASSANI, ERNANDES NAKAMURA, RENATO DE CAPUZZO, MURILO CATAFESTA DAS NEVES, PEDRO MAIA, KLECIUS LEITE FERNANDES, MARIO AUGUSTO DE CASTRO, MARIANNE YUMI NAKAI, LEANDRO LUONGO MATOS MATOS, FLAVIO CARNEIRO HOJAIJ, FÁBIO CAPELLI, GIULIANNO MOLINA DE MELO, LUCIO NOLETO, HELÁDIO FEITOSA E CASTRO, FATIMA CRISTINA MENDES DE MATOS, ALEXANDRE FERREIRA OLIVEIRA, RODRIGO NASCIMENTO PINHEIRO

**Affiliations:** 1 - Sociedade Brasileira de Cirurgia de Cabeça e Pescoço São Paulo - SP - Brasil; 2 - Sociedade Brasileira de Cirurgia Oncológica Rio de Janeiro - RJ - Brasil

**Keywords:** Thyroidectomy, Thyroid Gland, Surgical Oncology, Head and Neck Neoplasms, Tireoidectomia, Glândula Tireoide, Oncologia Cirúrgica, Neoplasias de Cabeça e Pescoço

## Abstract

The Brazilian Society of Surgical Oncology and the Brazilian Society of Head and Neck Surgery developed a technical consensus on hierarchical coding in thyroid surgery, considering the role of the Brazilian Hierarchical Classification of Medical Procedures in determining appropriate codes. A panel of 40 specialists - recognized academic and clinical leaders from across Brazil - assessed the applicability of procedure codes for common thyroid surgeries and prepared the current consensus. Deliberations were conducted via electronic voting among 37 participants, and consensus was defined as agreement by at least 80%. Scenarios included total thyroidectomy for benign and malignant disease and resection of substernal goiter. The analysis covered codes for laryngeal nerve exploration, neurophysiological monitoring, carotid artery branch ligation, biopsy, parathyroid reimplantation, lymphadenectomy, and neck dissection. Consensus supported the use of the following codes: nerve exploration (83.3%), neurophysiological monitoring (97.3%), biopsy and parathyroid reimplantation (89.2%), cervical lymphadenectomy (89.2%), and unilateral or bilateral neck dissections (97.3% and 94.6%, respectively). No consensus was reached on including carotid artery branch ligation or adding a partial thyroidectomy code for unilateral substernal goiter; therefore, these codes were not validated. The current consensus provides clear, objective guidance on hierarchical coding for thyroid surgery based on technical, scientific, and ethical criteria. It is intended to support attending physicians and auditors by promoting consistency, transparency, and reduced conflict in clinical and administrative settings.

## RESUMO

A Sociedade Brasileira de Cirurgia Oncológica e a Sociedade Brasileira de Cirurgia de Cabeça e Pescoço elaboraram um consenso técnico sobre a codificação hierarquizada na cirurgia da tireoide, considerando o papel da Classificação Brasileira Hierarquizada de Procedimentos Médicos no enquadramento das indicações para cada cirurgia. O documento foi elaborado por um colegiado de 40 especialistas, reconhecidas lideranças de representatividade acadêmica e assistencial, provenientes de diferentes regiões do país, que avaliaram a pertinência dos códigos aplicáveis às principais cirurgias da tireoide. As deliberações foram realizadas por meio de votação eletrônica entre 37 participantes, sendo o consenso estabelecido quando se atingiu concordância ≥ 80%. Foram avaliados cenários de tireoidectomia total para doenças benignas, malignas e para ressecção de bócio mergulhante, incluindo códigos de exploração de nervos laríngeos, monitorização neurofisiológica, ligadura de ramos da carótida, biópsia e reimplante de paratireoide, linfadenectomia e esvaziamentos cervicais. Houve consenso favorável à pertinência dos seguintes códigos: exploração de nervos (83,3%), monitorização neurofisiológica (97,3%), biópsia e reimplante de paratireoide (89,2%), linfadenectomia cervical (89,2%) e esvaziamentos uni ou bilaterais (97,3% e 94,6%), ratificando a utilização destes códigos. Não houve consenso para inclusão do código de ligadura da carótida e/ou ramos nem para agregar um código de tireoidectomia parcial em casos de bócio mergulhante apenas unilateral, afastando a validação destes. O consenso fornece normatização clara e objetiva para a codificação hierarquizada nas cirurgias da tireoide, fundamentada em critérios técnico-científicos e éticos. Destina-se a orientar médicos assistentes e auditores, promovendo uniformidade, transparência e redução de conflitos na prática clínica e administrativa.

## INTRODUCTION

Since its founding in 1951, the Brazilian Medical Association (AMB) has led efforts to guide physician fee billing in Brazil. Early guidance addressed only minimum fees and lacked standardization or hierarchy. The AMB first published a coded schedule of medical procedures in April 1967 - the Medical Fee Schedule - which was initially rejected by the National Department of Social Security^1^.

Over the following decades, the schedules underwent periodic revisions but continued to draw criticism for the absence of standardized measures of procedural complexity and execution time. Adoption also remained limited because many health insurers chose to use their own fee schedules.

Substantial progress came in 2003 with the first edition of the Brazilian Hierarchical Classification of Medical Procedures (CBHPM). This achievement resulted from collaboration among Brazil’s leading medical entities, including the AMB, the Federal Council of Medicine (CFM), the National Federation of Physicians, state-level organizations, and medical specialty societies^2^. Several health insurers adopted the CBHPM, and it was recognized by the National Regulatory Agency for Private Health Insurance and Plans (ANS). Today, the CBHPM is widely used in the supplementary health system for procedure requests and billing as well as for auditing, planning, and management. Codes require periodic review and updates to incorporate new procedures and technologies, reflecting ongoing advances in medical practice. Under CFM Resolution No. 1.673/2003, medical specialty societies and the AMB have exclusive authority to interpret their respective codes.

The hierarchical structure is central to determining medical fees, yet it can hinder fair compensation and contribute to health care-related litigation. A core issue is that every operation consists of a sequence of steps, each of which adds time and risk. These steps should generate codes proportional to the primary procedure.

Because medical specialty societies and the AMB hold exclusive authority to interpret these codes, many societies have issued guideline manuals for hierarchical coding of surgical procedures. These manuals require regular updates and serve as reference tools for physicians, particularly health-insurance auditors. Under Article 8 of CFM Resolution No. 1.614/2001 and Articles 52, 94, and 97 of Chapter XI of the current Brazilian Code of Medical Ethics (CFM Resolution No. 2217/2018), auditors may not deny or alter procedures requested by the attending physician. Misinterpretations - by attending physicians or auditors - often necessitate the involvement of a medical board to resolve disagreements regarding technical assistance, as supported by ANS Normative Resolution No. 424/2017.

The current document results from a collaboration between the Brazilian Society of Surgical Oncology (SBCO) and the Brazilian Society of Head and Neck Surgery (SBCCP). It presents a consensus that guides which codes may be requested and approved under hierarchical coding for thyroid surgery. The outcome is a clear, objective framework of applicable codes for the proposed procedures, serving as the definitive standard for arbitration to be observed by attending physicians and medical auditors.

## MATERIALS AND METHODS

The surgical specialty societies responsible for interpreting procedure codes and defining the hierarchical structure for thyroid surgery conceived and developed the current consensus. The SBCO and SBCCP established an institutional collaboration to convene a panel of experts guided by technical and scientific rigor and strict adherence to ethical principles. The group comprises recognized leaders in their fields, including faculty with master’s and doctoral degrees from public and private universities and preceptors affiliated with leading specialist-training institutions. The panel was intentionally structured to ensure representation from across Brazil. The selection process sought to avoid conflicts of interest that could compromise impartial evaluations or the application of scientific references.

A total of 40 specialists analyzed the codes eligible for use in the most common thyroid surgeries. Each code was discussed individually according to its indication. After detailed review and justification, each code and its corresponding recommendation was put to a vote by 37 specialists on the SurveyMonkey® platform, using a simple yes/no response. Consensus - and thus a formal recommendation - was defined as agreement by at least 80% of participants.

The committee used the 2022 edition of the CBHPM3 as the primary reference to frame the core questions, together with the Coding Guidelines Manuals for Head and Neck Surgery4 and Surgical Oncology5. The committee also conducted a critical review of relevant literature indexed in PubMed and SciELO, resulting in practical recommendations for hierarchical coding within Brazil’s health care system.

Three primary codes were initially evaluated as eligible for inclusion in surgical requests to determine appropriate coding for total thyroidectomy in benign conditions; 3.14.03.12-3 Surgical nerve exploration, 2.02.02.04-0 Intraoperative neurophysiological monitoring, 3.09.06.21-0 Carotid artery and/or branch ligation. Although not part of the initial set, two additional codes may be justified and added to the total procedure when performed and documented appropriately in the operative report; 3.02.14.01-7 Parathyroid biopsy, and 3.02.14.03-3 Parathyroid reimplantation. These two codes are not routine and should not be requested in advance.

For total thyroidectomy in cancer cases, the following codes were proposed; 3.09.14.05-1 Cervical lymphadenectomy (for suspicious lymph node assessment with frozen section biopsy, per level), 3.02.12.03-0 Unilateral neck dissection, 3.02.12.04-9 Bilateral neck dissection. Finally, the following code may be included either as an additional code or as a standalone code, depending on clinical necessity; 3.02.13.02-9 Resection of substernal goiter via cervicothoracic approach.

### Total thyroidectomy for benign diseases

In cases of total thyroidectomy for benign diseases, patients are candidates for removal of the thyroid gland. This procedure involves glandular dissection, surgical exploration, and preservation of the vagus and laryngeal nerves. Intraoperative neurophysiological monitoring and ligation of the superior thyroid arteries (branches of external carotid artery) aid in this process. Using code 3.02.13.05-3: Total thyroidectomy is mandatory. However, adding other procedure codes to total thyroidectomy has generated significant controversy, leading to disagreements between attending physicians and health insurance audit services. These codes are reviewed below.

#### Regarding code 3.14.03.12-3: Surgical nerve exploration

Surgical nerve exploration involves the external dissection of a nerve and its isolation from adjacent structures. This allows for direct intervention on anatomically associated organs, such as biopsies, grafts, transpositions, resections, or nerve releases. In thyroidectomy, this procedure has become essential in reducing complications and improving functional outcomes related to voice, respiration, and swallowing. Three nerves are directly involved in thyroid gland surgery: the recurrent laryngeal nerve (inferior laryngeal), the external branch of the superior laryngeal nerve, and the vagus nerve. These nerves may exhibit significant anatomical variations, so careful dissection while preserving these structures plays a major role in reducing rates of transient or permanent laryngeal paralysis and tracheostomy^6^ ^,^ ^7^. Such outcomes are not only serious complications for the patient, but they also increase treatment costs by prolonging hospital stays and requiring additional therapies, such as speech therapy or surgical procedures for voice rehabilitation. Therefore, neuromonitoring is fully justified.

Despite the growing recognition of the importance of identifying the laryngeal nerves, less experienced or specialized surgeons historically performed subtotal thyroidectomy more often. This surgical approach involves removing most of the thyroid gland without dissecting the laryngeal nerves, aiming to minimize complications such as nerve or parathyroid injury. The main downside, however, is incomplete resection of thyroid tissue. Since the early 21st century, subtotal thyroidectomy has fallen out of favor due to its significantly higher recurrence and reoperation rates without reducing permanent complication rates compared to lobectomy or total thyroidectomy^8^ ^-^ ^10^. Currently, the gold standard technique involves removing all thyroid tissue - either through lobectomy in unilateral cases or total thyroidectomy - along with a proven, beneficial safety procedure: exploration and dissection of laryngeal nerves^11^.

Surgical nerve exploration is not just another step in thyroidectomy; it is a procedure in its own right that requires more time, technical expertise, and resources.

The 2015 American Thyroid Association (ATA) Guidelines strongly recommend identifying, dissecting, releasing, and isolating the laryngeal nerves during both total and partial thyroidectomy, considering these steps essential to the best surgical practice^12^.

Question 1 - Is the use of code 3.14.03.12-3: Surgical nerve exploration, appropriate?

Answer 1 - Yes. According to the SurveyMonkey^®^ results, 30 out of 36 experts (83.33%) participating in this task force agreed that code 3.14.03.12-3: Surgical nerve exploration is appropriate to be requested in conjunction with the total thyroidectomy code. Only six (16.67%) were against it, and one expert abstained.

The final evaluation concluded that requesting this procedure alongside total thyroidectomy is appropriate.

While identifying, dissecting, releasing, and isolating the laryngeal nerves is considered part of the best surgical practices for thyroidectomy, this step should be considered additional operative time and counted as a separate procedure. 

#### Regarding code 2.02.02.04-0: Intraoperative neurophysiological monitoring

While some studies have not demonstrated a reduction in laryngeal nerve injury rates with the use of intraoperative neurophysiological monitoring (IONM), most of these studies are considered inadequate due to the low incidence of such complications. These complications range from permanent paralysis (1.1%) to transient paralysis (11%)^13^. Despite the low incidence of these events, they are dramatic for affected individuals, especially in bilateral cases, which may require a tracheostomy. Thus, the ATA recommends considering IONM, at minimum, to facilitate nerve identification and confirm neural function during thyroid surgeries^12^. This recommendation is especially important because 89% of nerve injuries result from traction on the neural structure^14^.

Four large-cohort, high-quality studies support the safety benefits of IONM in thyroidectomy: Kim et al. analyzed 17,610 thyroidectomies and found that IONM use was associated with a lower risk of recurrent laryngeal nerve injury ^13^; Wilhelm et al. reported in a cohort of 5,446 patients that routine IONM was independently associated with fewer complications, lower nerve-injury rates, and higher rates of same-day discharge^15^; in a European multicenter registry study, Staubitz et al. (EUROCRINE^®^ Council) evaluated more than 8,751 thyroidectomies and showed that IONM facilitates strategy adjustments to prevent bilateral vocal cord paralysis - thereby avoiding tracheostomy - and is associated with lower risks of nerve injury and vocal cord paralysis^16^; Clinically, bilateral laryngeal paralysis after thyroidectomy remains one of the most serious concerns when both lobes must be addressed; if unilateral paralysis is detected intraoperatively, IONM allows the surgeon to defer contralateral lobe resection to reduce the risk of airway compromise and emergency tracheostomy - a concern underscored by the systematic review and meta-analysis by Pardal-Refoyo and Ochoa-Sangrador^17^. Abdelhamid & Aspinall analyzed data from 42,341 patients and found that IONM was associated with a decreased risk of recurrent laryngeal nerve injury; they recommended its routine use during thyroid surgery^18^. 

Question 2 - Is the use of code 2.02.02.04-0: Intraoperative neurophysiological monitoring, appropriate?

Answer 2 - Yes. Among the 37 specialists who participated via SurveyMonkey^®^, 36 (97.30%) responded that code 2.02.02.04-0: Intraoperative neurophysiological monitoring is appropriate to be added to total thyroidectomy, establishing a strong consensus. Only 1 participant disagreed. 

#### Regarding code 3.09.06.21-0: Carotid artery and/or branch ligation

Ligation of external carotid artery branches is frequently required due to their anatomical proximity to key structures in the head and neck region, such as the thyroid gland, cervical lymph nodes, and muscles. This technique is well-established and supported by guidelines from AMB and other professional societies such as SBCCP and SBCO. The thyroid gland is highly vascularized, receiving blood primarily from the superior thyroid arteries (first branch of the external carotid artery) and inferior thyroid arteries (branches of the thyrocervical trunk)^19^.

Standard thyroidectomy techniques are well described in research, with refinements over the years contributing to improved safety and efficacy by reducing morbidity. Since the 1907 study by Halsted & Evans^20^, meticulous capsular dissection and avoidance of direct ligation of the superior thyroid artery have played a prominent role in minimizing complications such as permanent hypoparathyroidism and nerve injury. This is particularly important because the superior thyroid artery lies in close proximity to the external branch of the superior laryngeal nerve^21^, which, if injured, can compromise pitch modulation during phonation. For a long time, thyroidectomy was performed without direct ligation of the superior thyroid artery trunk to reduce this complication^22^.

However, with the increased technical expertise of specialized surgeons and advancements such as routine IONM, the incidence of injuries to the external branch of the superior laryngeal nerve has decreased significantly. Moreover, massive ligation of the upper thyroid pole has disadvantages such as increased bleeding risk^23^ and potential incomplete resection of thyroid, neoplastic, or lymphatic tissue. Some authors argue that ligation of the superior thyroid artery (a branch of the carotid artery) is currently justified due to better bleeding control and prevention of hematomas^24^ ^,^ ^25^, as well as facilitating complete dissection of the upper thyroid pole and removal of all thyroid tissue.

The main point of controversy lies in whether ligation of the superior thyroid artery should be considered an additional surgical step or whether it is, indeed, a routine component of thyroidectomy.

Question 3 - Is the use of code 3.09.06.21-0: Carotid artery and/or branch ligation appropriate during total or partial thyroidectomy? 

Answer 3 - No. Final SurveyMonkey^®^ results showed that this code remains highly controversial, with 19 specialists (51.35%) in favor and 18 (48.65%) opposed. As this did not reach the required consensus threshold, code 3.09.06.21-0: Carotid artery and/or branch ligation is considered not appropriate to be added to the thyroidectomy procedure and should be viewed as an inherent component of the surgery.

#### Regarding codes 3.02.14.01-7: Parathyroid biopsy and 3.02.14.03-3: Parathyroid reimplantation

During thyroidectomy, the surgical team may occasionally face uncertainty in distinguishing parathyroid glands from adjacent tissues, which may resemble them - such as lymph nodes, fat fragments, or even lymphatic metastases. In some cases, a biopsy may be necessary to assist in decision-making, as parathyroid glands should be preserved.

This scenario is especially relevant in thyroid cancer cases, where a parathyroid gland may be inadvertently resected along with central compartment lymphoadipose tissue during neck dissection. In such situations, the parathyroid gland must be reimplanted, and care should be taken to avoid mistakenly implanting neoplastic tissue.

The SBCO Guidelines on Indications and Technical Aspects of Cervical Lymph Node Dissection in malignant thyroid tumors recommend parathyroid biopsy before the gland is reimplanted^26^. Furthermore, both the SBCO and the Brazilian College of Surgeons recommend that parathyroid glands that are clearly devascularized or inadvertently removed with the surgical specimen should be properly prepared and reimplanted to prevent and manage post-thyroidectomy hypoparathyroidism^27^.

Question 4 - Is it appropriate for these codes to be added in the postoperative surgical report if the procedures are required during surgery?

Resposta 4, sim. A maioria dos especialistas foi favorável a adição dos códigos 3.02.14.01-7 (Biopsia da paratireoide) e 3.02.14.03-3 (Reimplante de paratireoide) em relatório pós-operatório, nos casos de necessidade para a realização destes procedimentos durante a tireoidectomia. O consenso foi atingido com 33 participantes (89.19%) respondendo sim ao Survey Monkey^®^, e com apenas 4 votos contrários (10.81%). Portanto os códigos são pertinentes de somatório a tireoidectomia em relatório pós-operatório justificado.

### Total Thyroidectomy for Malignant Diseases

Besides the codes previously discussed, 3 other codes are frequently required to ensure appropriate treatment of thyroid cancer, especially when lymph node metastatic involvement is suspected or confirmed.

#### Regarding code 3.09.14.05-1: Cervical lymphadenectomy (for evaluation of suspicious lymph nodes with intraoperative frozen section, by level)

Cervical lymphadenectomy is a surgical procedure performed to remove lymph nodes from the neck, indicated for evaluating nodal metastases in head and neck cancers - in this case, specifically thyroid neoplasms. It is a more targeted, selective procedure involving the removal of 1 or a few isolated lymph nodes. It must be differentiated from neck dissection, a broader procedure involving the removal of all lymph nodes in 1 or more neck levels.

During oncologic thyroidectomy, inspection of the central compartment (level VI) is a key step, since the rate of occult metastases in the central neck lymph nodes exceeds 50%, even among patients with papillary microcarcinoma and no preoperative clinical or imaging evidence of lymph node involvement^28^ ^,^ ^29^.

When metastatic involvement is suspected, a frozen section biopsy can be used - a rapid intraoperative exam that enables prompt analysis of the removed lymph nodes. However, there is ongoing debate regarding the clinical relevance of micrometastases (metastases ≤2mm), which may significantly increase the overall metastatic burden when counted.

While therapeutic cervical lymphadenectomy is commonly performed, prophylactic central compartment dissection is not recommended for patients with well-differentiated thyroid carcinoma T1 or T2 without clinically detectable metastases, as most high-quality evidence has failed to demonstrate a reduction in local recurrence rates, while showing an increased risk of complications.

Prophylactic central neck dissection may be indicated in advanced papillary carcinoma (T3 or T4) or when metastases are present in lateral neck levels (II to V), if it is important for planning adjuvant therapies12,26,30, and also in cases of medullary or anaplastic thyroid carcinoma^31^ ^,^ ^32^.

Cervical lymphadenectomy is listed in the CBHPM table by level and may be requested per indicated and performed level. 

Question 5 - Is the use of code 3.09.14.05-1: Cervical lymphadenectomy (for the evaluation of suspicious lymph nodes with intraoperative frozen section, per level), appropriate?

Answer 5 - Yes. Consensus was also reached in favor of this code’s appropriateness, with 33 participants (89.19%) responding “yes” on SurveyMonkey^®^, and only 4 participants (10.81%) opposing. Therefore, code 3.09.14.05-1: Cervical lymphadenectomy is considered appropriate and may be added to the thyroidectomy when justified according to the clinical and surgical context.

#### Regarding code 3.02.12.03-0: Unilateral neck dissection

Neck dissection is a surgical procedure that consists of compartmental resection of cervical lymph nodes in patients with head and neck malignancies. First described by Jawdyński in 1888 and later popularized by Crile in the early 20th century, the technique has been continuously refined alongside the evolution of scientific understanding of tumor biology and the development of new medical technologies^33^.

The systematization of neck dissection is based on the division of cervical lymph nodes into 6 anatomical levels^34^. Level I - Submandibular group, Level II - Upper jugular chain, Level III - Middle jugular chain, Level IV - Lower jugular chain, Level V - Posterior triangle nodes (behind the sternocleidomastoid muscle), Level VI - Central compartment (prelaryngeal, pretracheal, paratracheal, and recurrent nodes up to the brachiocephalic vessels, subdivided into right and left)^35^.

In the context of thyroid carcinomas, neck dissection is indicated only for therapeutic purposes, either when there is preoperative or intraoperative evidence of lymph node metastases, or electively in very specific situations. Elective dissection of lateral neck chains is not indicated.

The 8th edition of the American Joint Committee on Cancer TNM staging system categorizes patients with thyroid cancer based on regional lymph node staging (N category) as follows; N0 - No clinical evidence of metastasis in nearby lymph node, N1a - Metastasis in Level VI (central compartment - right or left), N1b - Metastasis in Levels I-V (lateral compartments) or retropharyngeal lymph nodes^36^. According to the most recent ATA guidelines, therapeutic neck dissection performed concurrently with thyroidectomy is indicated for patients with preoperative clinical evidence of nodal metastases (N1a or N1b). For N1a, resection should include prelaryngeal, pretracheal, and at least the ipsilateral paratracheal nodes (on the same side as the primary tumor). Contralateral paratracheal dissection may be considered. For N1b, dissection should encompass Levels II-V on the affected side, regardless of which individual level is involved. Elective dissection of the central compartment (Level VI) in patients with N0 nodal status may be considered in cases of locally advanced differentiated thyroid carcinoma (T3 or T4), medullary thyroid carcinoma, or anaplastic thyroid carcinoma^12^ ^,^ ^26^ ^,^ ^31^.

When code 3.02.12.03-0: Unilateral neck dissection is used, the attending surgeon indicates that Levels II-V on one side of the neck will be dissected therapeutically at the time of thyroidectomy, in accordance with established clinical guidelines. The 2022 CBHPM specifically reserves code 3.02.12.05-7 for central compartment dissection (Level VI), which must indicate laterality (right or left).

Question 6 - Is the use of code 3.02.12.05-7: Central compartment dissection (specific side), appropriate?

Question 7 - Is the use of code 3.02.12.03-0: Unilateral neck dissection (Levels II-V), appropriate?

Answer to Questions 6 and 7 - Yes. SurveyMonkey® responses regarding the therapeutic dissections described in Questions 6 and 7 showed strong consensus in favor of adding codes 3.02.12.05-7: Central compartment dissection (specific side) and 3.02.12.03-0: Unilateral neck dissection (Levels II-V) to the surgical procedure when clinically indicated. Consensus was reached with 36 participants (97.30%) voting “yes” and only 1 voting against.

#### Regarding code 3.02.12.04-9: Bilateral neck dissection

Although the principles of cervical dissection have already been addressed in the previous section, it is worth noting that bilateral procedures may be required under certain conditions. In the presence of N1a nodal status, central compartment dissection on both the right and left sides is recommended. Even in the absence of clinical evidence of metastasis, this dissection is indicated for well-differentiated thyroid tumors classified as T3 or T4, as well as for all cases of medullary or anaplastic thyroid carcinoma.

When N1b nodal status are present, the dissection should include Levels II-V on the affected side, and must be extended bilaterally when lymph node involvement is present on both sides of the neck. In medullary thyroid carcinoma, elective bilateral dissection of Levels II-V may also be considered in patients with preoperative calcitonin levels above 20 pg/mL^12^ ^,^ ^26^ ^,^ ^31^.

Question 8 - Is the use of code 3.02.12.04-9: Bilateral neck dissection, appropriate?

Answer 8 - Yes. With regard to code 3.02.12.04-9: Bilateral neck dissection, the SurveyMonkey^®^ responses showed strong support for adding this code when clinically indicated, with 35 participants (94.59%) voting in favor and only 2 votes against. Therefore, this code is considered appropriate for use when justified. 

#### Resection of Substernal Goiter

There is no universally accepted definition of substernal goiter in literature. However, it is generally understood as the extension of thyroid tissue into the mediastinum^37^ ^-^ ^39^. In most cases, the descent into the mediastinum results from enlargement of the inferior portion of the thyroid lobe, which may occur unilaterally or bilaterally. In some cases, it may originate from ectopic thyroid tissue located at the mediastinal inlet, with no anatomical connection to the thyroid gland and with distinct vascularization^39^ ^,^ ^40^. In over 95% of cases, the initial diagnosis is benign^41^.

The extent of surgery depends on the type of lesion that led to surgical indication. Total thyroidectomy is not always required when the disease is unilateral^37^ ^,^ ^42^. In some cases, only resection of the ectopic thyroid tissue with mediastinal extension may be needed. When resection of the substernal component is performed, the appropriate procedural code is 3.02.13.02-9: Resection of substernal goiter via cervicothoracic approach. Other codes may be added as appropriate, depending on the associated surgical procedures performed.

Question 9 - Does every resection of a substernal goiter imply a total thyroidectomy?

Answer 9 - No. This question also reached consensus, with 83.78% (31 participants) stating that not every substernal goiter resection requires total thyroidectomy. Only 6 participants disagreed with this statement.

This consensus is important because it clarifies that code 3.02.13.02-9 Resection of substernal goiter via cervico-thoracic approach may be requested specifically for the resection of the portion or lobe responsible for the substernal extension. It does not necessarily imply complete thyroid removal, since one of the thyroid lobes may remain healthy and unaffected.

Question 10 - When the substernal goiter is unilateral and there is a separate indication for contralateral lobectomy, is it appropriate to add code 3.02.13.04-5: Partial thyroidectomy?

Answer 10 - No. Although most participants agreed that resection of a substernal goiter does not necessarily imply a total thyroidectomy, consensus was not reached regarding the addition of a separate code for removal of the non-substernal lobe. In the SurveyMonkey® results, 12 participants (32.43%) disagreed with the possibility of combining code 3.02.13.04-5: Partial thyroidectomy with the code for resection of substernal goiter under these circumstances. Therefore, when code 3.02.13.02-9: Resection of substernal goiter via cervicothoracic approach is requested and complete thyroid removal is indicated, this should be the only thyroid-related code used, and the codes for total or partial thyroidectomy should not be combined with it. 

Question 11 - During substernal goiter resection, are the codes associated with thyroidectomy also appropriate?

Answer 11 - Yes. This question also reached consensus, with 31 participants (83.78%) confirming the appropriateness of applying all thyroidectomy-related codes to substernal goiter resection, according to the SurveyMonkey^®^ responses. Therefore, all codes deemed appropriate for thyroidectomy should also be considered appropriate for resection of substernal goiter.

**Figure 1:**
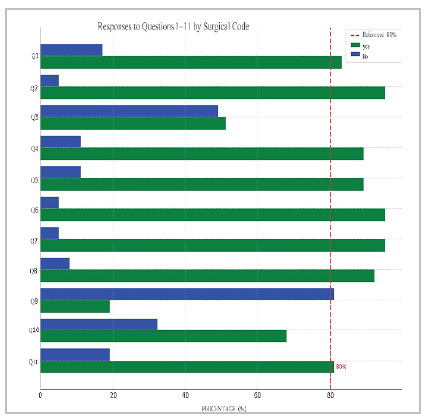
Overall voting result.

## CONCLUSION

The current technical review, the result of collaboration between SBCO and SBCCP, represents an important milestone in the process of standardizing the hierarchical coding of surgical procedures related to thyroidectomy in Brazil. By establishing clear, evidence-based guidelines for the inclusion of complementary procedure codes in thyroidectomy, the current consensus contributes to reducing conflicts between attending physicians and auditors, strengthening transparency, and appropriately valuing the technical complexity of these interventions.

The consensus methodology - based on broad participation from experts and a critical analysis of both national and international guidelines and literature - adds legitimacy and robustness to the proposed recommendations. Validating the relevance of codes such as surgical nerve exploration, intraoperative neurophysiological monitoring, and various types of cervical dissections reflects the recognition of the technical diversity that may be required in each clinical scenario, while respecting procedural complexity.

The current guideline, backed by consensus, establishes the appropriateness of certain codes for audit and regulatory purposes. However, the final decision regarding which codes to request must remain under the responsibility of the attending surgeon, guided by technical-scientific criteria, the individual clinical context, and an ethical commitment to patient safety and good medical practice. By upholding physician autonomy while offering normative support, the current consensus significantly contributes to improve Brazil’s supplementary health system and to the protection of specialized medical practice.
